# Juvenile‐onset Huntington's disease – Spectrum and evolution of presenting movement disorders

**DOI:** 10.1002/acn3.52193

**Published:** 2024-09-06

**Authors:** Kathryn Yang, Vicente Quiroz, Amy Tam, Rasha Srouji, Ximena Villanueva, Claudia Amarales, Darius Ebrahimi‐Fakhari

**Affiliations:** ^1^ Movement Disorders Program, Department of Neurology, Boston Children's Hospital Harvard Medical School Boston Massachusetts USA; ^2^ Neurology Unit Hospital Carlos van Buren Valparaíso Chile

## Abstract

Juvenile‐onset Huntington's disease (HD) is a rare subset of HD with symptom‐onset before the age of 18. In contrast to the adult population, children present early‐on with behavioral, psychiatric, and cognitive symptoms, in addition to a diverse spectrum of movement disorders. This poses a distinct challenge in diagnosis and management. We here describe the spectrum of movement disorders, accompanied with detailed video recordings, in seven cases of juvenile‐onset HD. Our findings highlight early cognitive and behavioral symptoms, preceding motor symptoms. The diverse movement disorder phenotypes included dystonia, Parkinsonism, myoclonus, and chorea, findings which underscore the heterogeneity of presenting symptoms.

## Introduction

Huntington's disease (HD) is a progressive neurodegenerative disorder caused by abnormal CAG repeat expansions in the huntingtin gene.^1^ The resulting neuronal loss, predominantly in the basal ganglia and cerebral cortex, causes movement disorders, cognitive decline, and psychiatric concerns.^2^ Juvenile‐onset HD is rare, accounting for ~3%–10% of cases, and can present with symptoms that are less common in adult patients, such as epilepsy.^3^, ^4^, ^5^, ^6^, ^7^ Motor symptoms also diverge; chorea is less common, while mixed movement disorders of parkinsonism and dystonia are more prevalent.^4^, ^8^ Given the low number of reported cases, however, the full spectrum of movement disorders in children with HD remains incompletely understood. Here, we detail seven cases of juvenile‐onset HD and document presenting movement disorders.

## Patients and Methods

A retrospective review of clinical data, neuroimaging, and video recordings of children with a molecularly confirmed diagnosis of HD, evaluated between 2022 and 2024. This study was approved by the Institutional Review Board at Boston Children's Hospital (IRB‐P00044666).

## Results

The clinical and molecular data of seven patients with juvenile‐onset HD are detailed in Table 1. This cohort included three females and four males, with expanded CAG repeats ranging from 63 to 102, demonstrating only a limited correlation with age at symptom onset (*r* = −0.56, 95%CI [−0.93, 0.31]). The mean age at motor symptom onset was 10.4 ± 3.9 years (SD, range: 6–15 years), 12.0 ± 4.1 years (SD, range: 7–17 years) at molecular diagnosis, and 14.3 ± 3.2 years (SD, range: 9–18 years) at last follow‐up. Notably, five out of seven had developmental delay/intellectual disability, three out of seven later showed cognitive decline, and four out of seven and two out of seven were diagnosed with attention deficit/hyperactivity disorder (ADHD) and autism spectrum disorder (ASD), respectively. Behavioral and psychiatric manifestations were prevalent and severe. This included aggressive, impulsive, and self‐injurious behaviors (4/7), anxiety disorder (4/7), and major depressive disorder (2/7). In all patients except one, cognitive and behavioral symptoms preceded motor symptoms, often by several years. Sleep disorder was significant in one out of seven. Epilepsy was a late manifestation found in two patients. All patients presented with severe and progressive dysarthria, two out of seven developed dysphagia significant enough to warrant a gastrostomy tube.

**Table 1:** Summary of demographic data and clinical manifestations in a cohort of seven children with juvenile‐onset Huntington's disease.

Clinical picture							
Patient ID	Patient 01	Patient 02	Patient 03	Patient 04	Patient 05	Patient 06	Patient 07
Sex	Male	Female	Female	Female	Male	Male	Female
Age of motor symptom onset (years)	13	10	16	6	15	7	7
Age at molecular diagnosis	15	12	16	9	17	7	8
Age at last neurology follow‐up	18	15	16	15	16	11	9
CAG repeats	63	69	70	80	80	84	102
Inheritance pattern	Paternal	Paternal	Unknown	Maternal	Unknown	Paternal	Paternal
Motor contro							
Chorea		+	+	+		+	
Dystonia	+			+	+	+	+
Myoclonus	+	+		+		+	
Ataxia					+		
Tremor					+		
Stereotypies					+		
Hypo/bradykinesia	+			+	+		+
Postural instability	+			+		+	
Rigidity				+			+
Phonatory dysfunction	+	+		+	+	+	+
Cognitive symptoms							
History of developmental delay		+	+	+		+	
Intellectual disability			+	+			
Cognitive decline	+		+	+		+	
Attention‐deficit/hyperactivity disorder		+	+			+	+
Autism spectrum disorder					+	+	
Behavioral symptoms							
Aggressive behavior			+	+	+	+	
Self‐injurious behavior			+	+	+		
Anxiety		+		+		+	+
Depression		+	+				
Impulsivity			+	+		+	
Sleep disorder				+			
Other symptoms							
Epilepsy	+			+			
Eye movement disorder		+			+		+

The spectrum of movement disorders at initial presentation included dystonia (5/7), parkinsonism (4/7), myoclonus (4/7), and chorea (4/7). Chorea developed along the disease course but was rarely an initial concern. Ataxia, tremor, and stereotypies were seen in one individual. The characteristics and evolution of different movement disorders are detailed in case vignettes below, with accompanying supplementary videos.

**Figure 1:**
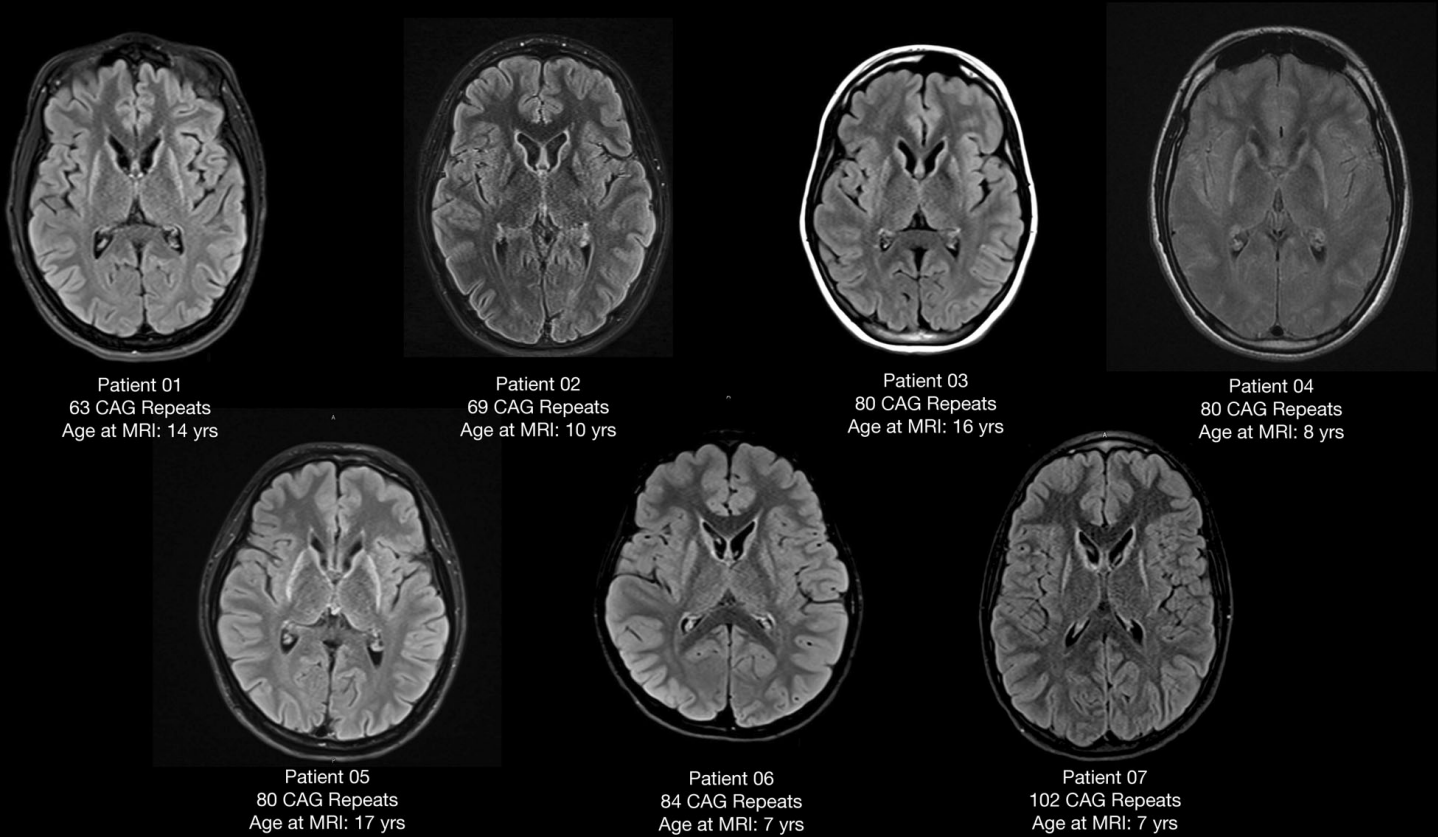
Brain MR imaging with representative axial T2‐FLAIR images demonstrating the universal feature of atrophy and signal prolongation in the caudate and putamen bilaterally.

Brain MR imaging was typically performed around the onset of motor symptoms, showing significant volume loss and signal changes within bilateral caudate and putamen (Fig. 1).

### Patient #1 (Video S1)

This 18‐year‐old male (63 CAG repeats) of Arab background had an extensive paternal family history of HD and presented at 13 years of age with dystonic posturing of his distal legs, segmental myoclonus of the upper body, motor apraxia with fine motor impairment, and daytime fatigue. Video EEG showed epileptic and non‐epileptic myoclonus with interictal generalized discharges, leading to therapy with valproate, with partial response. Over time, motor function and cognition declined significantly, with worsening of myoclonus, development of parkinsonism (bradykinesia, hypomimia, and hypophonia), cervical dystonia, dysarthria, and a decline in processing speed and memory. Treatment with levodopa/carbidopa improved bradykinesia. At the last follow‐up, no behavioral or psychiatric symptoms were noted.

### Patient #2 (Video S2)

This 15‐year‐old female (69 CAG repeats) of mixed European background had a paternal history of HD. The initial symptoms in early childhood included motor delay and apraxia, learning disability, ADHD, anxiety disorder, and depression. At 10 years, she developed an abnormal gait with difficulties in running and climbing stairs, along with a significant decline in academic performance. Neurological examination showed mild dysarthria, myoclonus of the neck and shoulders, and choreiform movements of both hands and right leg while walking. Over the course of 2 years, she developed severe dysarthria, slow horizontal saccades, restlessness with motor impersistence, and chorea of both arms. Symptoms responded partially to clonazepam and gabapentin.

### Patient #3

This 20‐year‐old Hispanic female (70 CAG repeats) had an unknown family history. Early psychiatric concerns included disruptive mood dysregulation disorder at the age of 10 years, followed by depression with self‐harm and suicide attempts. At 15 years, she began experiencing frequent tripping. Serial neurological examination noted subtle choreiform movements in her limbs.

### Patient #4 (Video S3)

This 13‐year‐old Hispanic female (80 CAG repeats) had a maternal history of HD. At 6 years of age, a decline in school performance was noted, initially attributed to anxiety, poor executive function, and impulsivity. Subsequent neurological examination was notable for mild dysarthria, generalized rigidity, and pyramidal signs. Over 2 years, she developed severe cervical dystonia with anterocollis, postural, and action‐induced dystonia of the upper limbs, and hypomimia. Symptomatic treatment included botulinum toxin injections, baclofen, levodopa/carbidopa, and pramipexole. At 11 years, risperidone was added due to aggressive behaviors, poor sleep, and rapid cognitive decline. At last follow‐up, clinical manifestations included generalized chorea, cervical dystonia, rigidity of the limbs, motor impersistence, gait impairment with frequent falls, and dysphagia necessitating gastrostomy tube dependence. At 12 years she was diagnosed with epilepsy.

### Patient #5 (Video S4)

This 18‐year‐old Asian male (80 CAG repeats) was adopted and had a limited family history. Concerns in childhood included motor apraxia, stereotypies, and persistent toe‐walking. A diagnosis of ASD was made at the age of 10 years, and self‐injurious behaviors such as head banging and hair pulling emerged. At 15 years, he developed a postural hand tremor and difficulties walking. Neurological examination showed mild dysarthria, motor stereotypies described as hand wringing, cervical dystonia with latero‐ and anterocollis, postural dystonia of the arms, and a fine postural and action tremor. At the last follow‐up examination ataxia, severe dysarthria, prominent abnormal saccades, dysdiadochokinesia, and mild lower extremity spasticity with pyramidal signs were noted.

### Patient #6 (Video S5)

This 12‐year‐old male (84 CAG repeats) of mixed racial background had a paternal history of HD. First concerns arose at 12 months with delayed developmental milestones. Independent walking was achieved at 18 months, and first words at 24 months. Diagnosed with ADHD, ASD, and learning disability, at 7 years he experienced a progressive decline in his gait with frequent falls. At last follow‐up, he had myoclonus of the proximal upper extremities, limb chorea during walking, segmental dystonia involving the upper limbs with painful hand spasms, and mild parkinsonism (mainly bradykinesia with hypomimia). Severe dysarthria led to complete loss of verbal communication, and he developed dysphagia with significant weight loss necessitating gastrostomy tube feeds. Quality of life was further impacted by anxiety and aggressive, self‐injurious, and impulsive behaviors. Treatment included a combination of risperidone, fluoxetine, clonazepam, clonidine, and tetrabenazine, with modest benefit.

### Patient #7 (Video S6)

This 9‐year‐old female (102 CAG repeats) of mixed European background had a paternal history of HD. Early developmental milestones were normal, but ADHD and generalized anxiety disorder were diagnosed at 5 years. Around 7 years, she was noted to have increased motor difficulties, particularly with running and climbing stairs. Speech declined rapidly due to dysarthria, rending her nonverbal and reliant on augmentative communication devices. Neurological examination at last follow‐up showed severe dysarthria and hypophonia, cervical and appendicular dystonia, parkinsonism (bradykinesia and mild rigidity of the limbs), and hypometric saccades. Levodopa‐carbidopa led to modest improvement of bradykinesia.

## Discussion

In this cohort, evaluated at tertiary care pediatric movement disorders clinics, the mean age at onset of motor symptoms was 10 years, with all patients having over 60 CAG repeats, in all but one through paternal transmission. Our results expand on previous studies that have highlighted the rapid progression and severe manifestations in juvenile‐onset HD.^4^, ^9^, ^10^, ^11^, ^12^ Notably, we document the occurrence of non‐motor symptoms long before onset of movement disorders. This includes developmental delays, learning disability, and a myriad of behavioral and psychiatric manifestations.

The rarity of juvenile‐onset HD complicates a timely diagnosis as many pediatric neurologists may not encounter it during their careers. Its atypical presentation compared to adult‐onset forms also makes it less likely to be considered in differential diagnoses.^7^

In our sample, the most common movement disorders were parkinsonism and dystonia, both seen in five of seven cases. On average, patients displayed more than three movement disorder phenomenologies, reinforcing the notion that juvenile‐onset HD commonly presents with a mixed movement disorder.^11^ Notably, Patients 1 and 7 exhibited predominantly motor symptoms with minimal to mild cognitive or behavioral concerns. In contrast, Patient 3 exhibited mainly psychiatric symptoms about 5 years before onset of chorea. This heterogeneity confirms prior observations in juvenile‐onset HD cohorts.^4^

Brain MR imaging provided an important diagnostic step in all cases within our cohort. Pronounced bilateral atrophy and T2 signal prolongation of the caudate and putamen should raise concern for HD and prompt molecular testing.^13^, ^14^

Treatment of juvenile‐onset HD focuses on interdisciplinary symptom management and quality of life. Children with HD are generally excluded from interventional trials, which poses a significant challenge, particularly in the setting of rapid disease progression.^15^

From a movement disorders perspective, it is essential to tailor symptomatic treatment to the presenting phenomenology with an understanding that movement disorders evolve rapidly and are often mixed. In our cohort, early manifestations usually included parkinsonism and dystonia, the latter typically progressing quickly. Myoclonus was also frequent, whereas chorea developed later. This general shift from hypo‐ to hyperkinetic movement disorders over time, although not universal, necessitates adaptation of treatment strategies. Synergies with the treatment for other disease manifestations, namely impulsive and aggressive behaviors or sleep disorder, should be explored given interventions such as benzodiazepines or dopamine‐depleting agents might ameliorate both.

Our study is limited by a relatively small sample size, short follow‐up, and inconsistency in use of standardized rating scales (e.g., the UHDRS or BFMDRS). Thus, the full spectrum and evolution of movement disorders in juvenile‐onset HD remains to be further delineated through larger prospective studies.

In conclusion, we highlight the unique phenotypic manifestation in juvenile‐onset HD, with a focus on movement disorder phenomenology, and demonstrate important differences when compared to adult‐onset HD.

## Author Contributions

K.Y., V.Q., and D.E.‐F. conceptualized and designed the study. K.Y., V.Q., and D.E.‐F. performed analyses. K.Y. drafted the original manuscript, with contributions from other authors. All authors contributed to the final draft of the manuscript.

## Conflicts of Interest

D. Ebrahimi‐Fakhari has received speaker honoraria from The Movement Disorders Society and the Taiwan Society of Child Neurology, and consulting fees from Guidepoint LLC, the University of Texas, the Telethon Foundation, and the German Center for Neurodegenerative Diseases (DZNE).

## Supporting information


Video S1.

Video S2.

Video S3.

Video S4.

Video S5.

Video S6.

## Data Availability

The data that support the findings of this study are available from the corresponding author upon reasonable request.
